# Biotrophy-necrotrophy switch in pathogen evoke differential response in resistant and susceptible sesame involving multiple signaling pathways at different phases

**DOI:** 10.1038/s41598-017-17248-7

**Published:** 2017-12-08

**Authors:** Supriyo Chowdhury, Arpita Basu, Surekha Kundu

**Affiliations:** Molecular and Applied Mycology and Plant Pathology Laboratory Department of Botany, University of Calcutta, 35, Ballygunge Circular Road, Kolkata, 700019 India

## Abstract

Infection stages of charcoal rot fungus *Macrophomina phaseolina* in sesame revealed for the first time a transition from biotrophy via BNS (biotrophy-to-necrotrophy switch) to necrotrophy as confirmed by transcriptional studies. Microscopy using normal and GFP-expressing pathogen showed typical constricted thick intercellular bitrophic hyphae which gave rise to thin intracellular necrotrophic hyphae during BNS and this stage was delayed in a resistant host. Results also show that as the pathogen switched its strategy of infection, the host tailored its defense strategy to meet the changing situation. Less ROS accumulation, upregulation of ROS signaling genes and higher antioxidant enzyme activities post BNS resulted in resistance. There was greater accumulation of secondary metabolites and upregulation of secondary metabolite-related genes after BNS. A total of twenty genes functioning in different aspects of plant defense that were monitored over a time course during the changing infection phases showed a coordinated response. Experiments using phytohormone priming and phytohormone inhibitors showed that resistance resulted from activation of JA-ET signaling pathway. Most importantly this defense response was more prompt in the resistant than the susceptible host indicating that a resistant host makes different choices from a susceptible host during infection which ultimately influences the severity of the disease.

## Introduction

Fungal plant pathogens can be classified as biotrophs, necrotrophs or hemibiotrophs based on their life-style and interaction with the host. Hemibiotrophic fungi represent the most interesting group of pathogens since they use sequential biotrophic and necrotrophic infection strategies to invade and colonize host plants^1^. Transition from the asymptomatic biotrophic phase, characterized by intercellular thick primary hyphae, to the destructive necrotrophic phase, characterized by thin filamentous secondary hyphae, is referred to as the biotrophy-necrotrophy switch (BNS). BNS is characteristic of typical hemibiotrophic fungi like *Colletotrichum sp* ^2^, *Phytopthora capsici* ^3^ and *Moniliophthora roreri* ^3–10^.

The BNS and modulation of plant defense in response to BNS is not well understood^4^. One important strategy for studying these dynamic but highly regulated host defenses is to observe gene expression patterns in the host^5^. There are only a handful of studies in hemibiotrophic fungi-host interaction^5^ most of these being the overall transcriptome analysis of the pathogen and/or the host^2–4,6–8^ without going into details of individual gene expression before, during and after BNS in the pathogen. The few existing studies at the transcriptional level do show that the host can tailor the defense response according to the changing phases of the pathogen^9–11^. There are only two preliminary studies that involve susceptible and resistant host during interaction with hemibiotrophic pathogens^12,13^.

Phytohormone signaling involving JA, ET or SA is an integral component of multilayered host-defense system against plethora of fungal pathogens. The lifestyle of a pathogen often dictates the host’s defense strategy and the pathogen may even manipulate hormonal cross-talk for successful colonization^14^. On the counter side plants adopt timely activation of suitable phytohormone signaling depending on the pathogen’s lifestyle so as to restrict the infection process^12^.

Sesame (*Sesamum indicum* L.), one of the most important oilseed crop is cultivated throughout the world in tropical and subtropical regions. Yet there is an absolute lack of any transcriptional study regarding molecular defense response of sesame against any biotic/abiotic stress. One of the major threats of global sesame production is the charcoal rot disease caused by *Macrophomina phaseolina* (Tassi) Goid^15,16^. With the recent availability genome sequence of sesame^17^, it has opened up an enormous scope for studying defense related gene regulation in response to stresses. Moreover, *M. phaseolina* has thus far been classified as a necrotrophic pathogen. In spite of our earlier report on *M. phaseolina* infection in sesame^15^, whether a brief biotrophic phase exists for the pathogen during initial infection stage was never investigated.

In the present study, during the early infection stages of *M. phaseolina* in sesame, we detected a lifestyle transition from biotrophy to necrotrophy which has been supported by microscopic and transcriptional data. We attempted to understand if the host defense response is tailored specifically to meet the changing strategies of the pathogen i.e. as the pathogen moves from a biotrophic phase through the BNS and finally to a necrotrophic phase. Along with microscopic and biochemical studies, in order to get an insight into the defense signaling against a hemibiotrophic fungi, we have investigated whether priming with different phytohormones evokes resistance. Moreover we attempted to investigate for the first time whether this dynamic molecular response of the host against a hemibiotrophic pathogen, is different in a resistant versus a susceptible host. The transcript levels of a total of 20 genes functioning in the different arms of defense signaling and changes in phytohormone levels have been monitored over a time course to get an insight into the different choices a resistant host makes to combat the infection which a susceptible host cannot.

## Results

### A distinct biotrophic phase is followed by necrotrophic phase in *Macrophomina phaseolina* during infection of sesame

Observation of *M. phaseolina* infection in sesame roots revealed a short yet distinct biotrophic phase followed by transition from biotrophy to necrotrophy (BNS phase) and then a prominent necrotrophic phase. The duration of each phase and its transition to next one varied considerably between the susceptible (VRI-1) and resistant (Nirmala) variety. The infection stages were studied using normal and transformed *M. phaseolina* expressing GFP.

**Figure 1:**
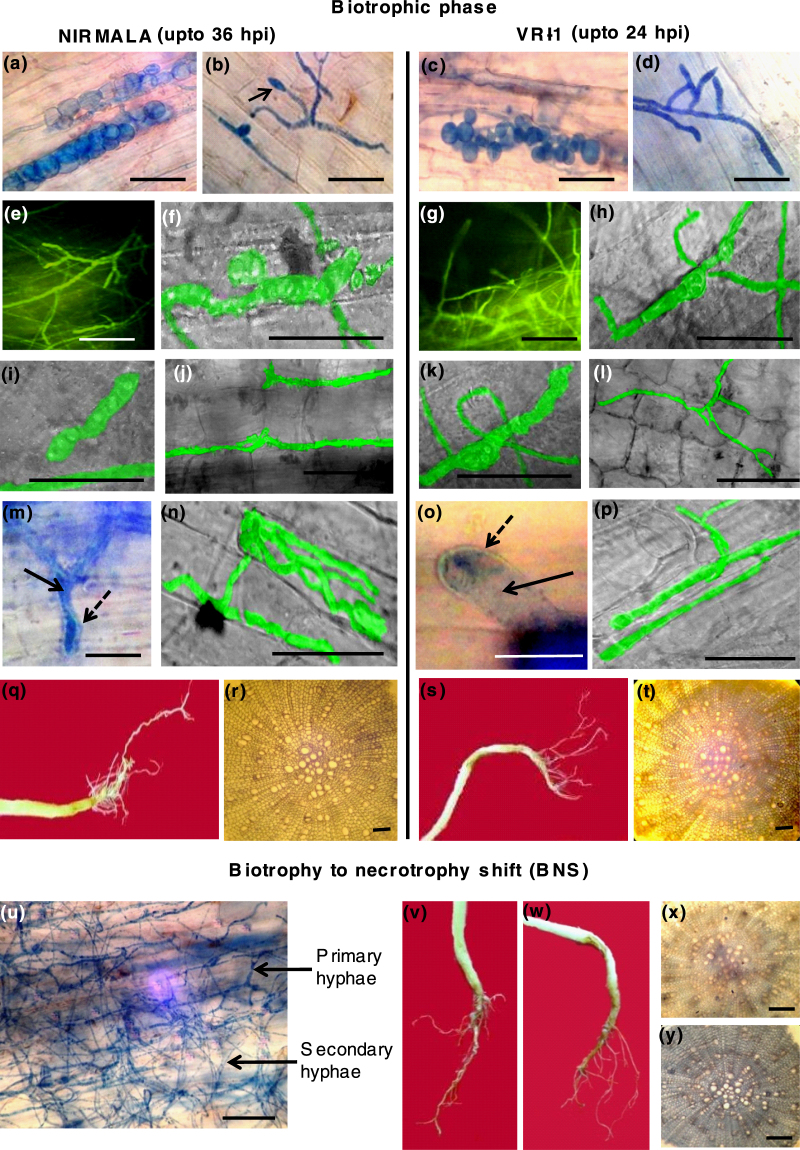
*Macrophomina phaseolina* infection of sesame shows a hemibiotrophic life cycle during infection of resistant (Nirmala) and susceptible (VRI-1) varieties of sesame. (**a**,**c**) Formation of intracellular vesicles in roots (3 hpi). (**b**,**d**) Hyphopodia arising from vegetative hyphae on sesame root surface during early biotrophic phase (3–6 hpi). (**e**,**g**) Confocal microscopy showing GFP-expressing runner hyphae on root surface of resistant variety Nirmala and susceptible VRI-1 (9–12 hpi). (**f**,**h**,**i**,**k**) confocal microscopy showing presence of typical constricted biotrophic hyphae within root tissues of both varieties (12 hpi). (**j**,**l**) GFP expressing intercellular biotrophic hyphae (16–24 hpi). (**m**,**o**) Typical intracellular double membrane-bound biotrophic hyphae during late biotrophic phase, solid arrow indicates biotrophic hypha and dashed arrow plant derived membranes (32–34 hpi for Nirmala, 20–22 hpi in VRI-1). (**n**,**p**) GFP expressing hyphae within a few host cells during late biotrophic phase. (**q**,**s**) External view of lower stem and root of Nirmala and VRI-1 during biotrophic phase showing no visible symptoms. (**r**,**t**) Cross-section of roots of two varieties during biotrophic infection phase showing no necrotrophy. (**u**) Transition from biotrophy to necrotrophic phase (BNS) was characterized by development of thin, secondary necrotrophic hyphae from primary biotrophic hyphae. (**v**,**w**) External view of lower stem and root Nirmala nad VRI-1 still showing little or no symptoms. (**x**,**y**) Cross section of roots during BNS with trypan blue staining. BNS was delayed in the resistant variety being observed at 36–38 hpi in Nirmala and at 24–26 hpi in VRI-1. (Bar = 50 µm).

At early biotrophic phase (3–4 hpi), typical aggregates of swollen vesicles consistent with biotrophic phase of other hemibiotrophic fungi^18^, were found in the root cortical tissues of both resistant variety (Fig. 1a) and susceptible variety (Fig. 1c). Associated with infection pegs, hyphopodia-like penetrating structure were visible at infection sites in both susceptible and resistant roots (Fig. 1b,d). Infection with GFP-expressing transformed *M. phaseolina* followed by confocal microscopy revealed root surface colonization by typical biotrophic runner hyphae^18^ at 9–12 hpi. (Fig. 1e,g). After penetration, the fungi produce typically constricted intracellular biotrophic primary hyphae in both varieties (Fig. 1f,h,i,k). Following penetration, the hyphae remained mostly intercellular, avoiding direct penetration of host cells in the root epidermis and cortical tissue at 16–24 hpi (Fig. 1j,l). Even in places where the hyphae did penetrate the host cells, the hyphal extension invaginated into the host plasma membrane thereby covering itself with the host membrane which is a typical strategy of host defense avoidance in biotrophic fungi (Fig. 1m,o). Development of double membrane intercellular primary hyphae took place during late hours of biotrophy, 32–34 hpi for Nirmala, 20–22 hpi in VRI-1. The biotrophic phase was also characterized by typical thick, biotrophic primary hyphae confined initially to infected epidermal cells (Fig. 1n,p).

During biotrophic phase, roots of both varieties appeared externally symptomless (Fig. 1q,s). The internal tissues also remained symptom-free with cross-sections of root showing no evidence of tissue necrosis up to 24 hpi in VRI-I and 36 hpi in Nirmala (Fig. 1r,t). The biotrophic infection phase was completed within 24 hrs in VRI-1 roots, but it continued up to 36–38 hrs in Nirmala.

Timing of transition from biotrophy to necrotrophic phase (BNS phase) of *M. phaseolina* was delayed considerably in the resistant variety compared to the susceptible one, being 36–38 hpi for Nirmala and 24 hpi for VRI-1. During BNS, majority of biotrophy-associated thick primary hyphae gave rise to thinner necrotrophy-associated secondary hyphae (Fig. 1u). Still no prominent symptoms were visible in stems and roots of the two varieties up to this particular phase (Fig. 1v,w,x,y).

**Figure 2:**
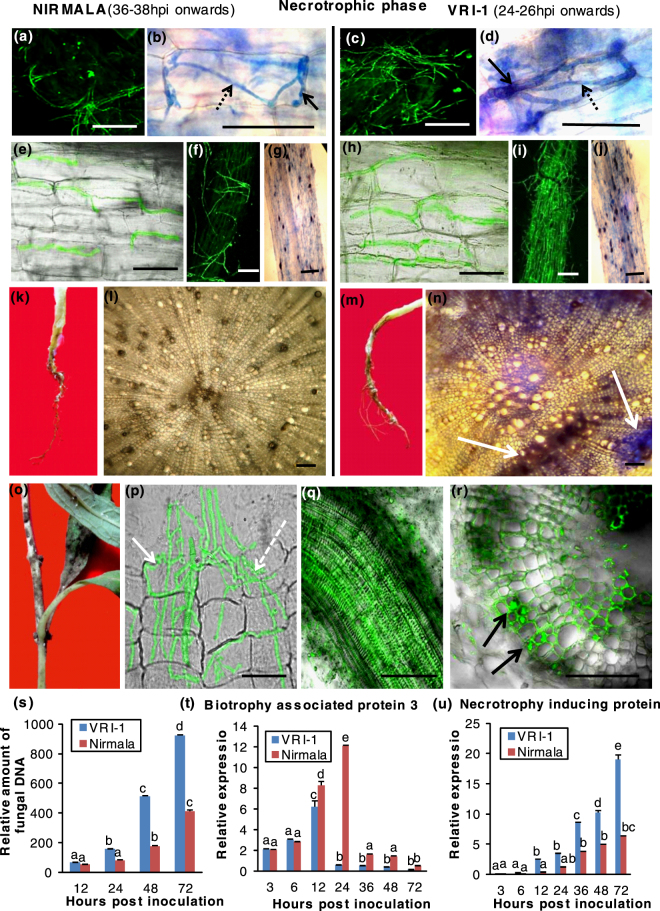
Necrotrophic phase of *Macrophomina phaseolina* infection in resistant sesame cultivar Nirmala (36–38 hpi onwards) and susceptible cultivar VRI-1 (24–26 hpi onwards). (**a**,**c**) Less extensive growth of GFP-expressing thin secondary hyphae in root tissues during necrotrophic phase in Nirmala (38 hpi) compared to VRI-1 (26 hpi). (**b**,**d**,**e**,**h**) Trypan blue staining and confocal microscopy using GFP-expressing mycelia show intercellular secondary necrotrophic hyphae (solid arrows) growing around the outline of the root cells and intracellular hyphae (dashed arrows) inside root cells in Nirmala and VRI-1. (**f**,**i**) Confocal microscopy showing scanty growth of GFP-expressing mycelia on root surface in Nirmala while in VRI-1 the root surface is covered by a mantle of fungal hyphae (72 hpi). (**g**,**j**) Less hyphal growth and microsclerotia (appearing as elongated black spots) on Nirmala roots compared to VRI-1 (96–120 hpi). (**k**,**m**) External symptoms of root in Nirmala (72 hpi) and VRI-1 (60 hpi) during early necrotrophic phase. (**l**,**n**) Trypan blue staining of root cross section showing less necrotrophic symptoms in Nirmala than VRI-1 at 96 hpi (black degenerated tissues shown by white arrow) (**o**) External symptoms on infected VRI-1 stem sowing typical charcoal rot symptom (120 hpi). (**p**) Confocal microscopy showing stem cortical cells heavily colonized with GFP-expressing secondary hyphae. Intercellular growth of hyphae is shown by solid arrow and intracellular growth by dashed arrow. (**q**,**r**) Longitudinal section of stem showing a group of xylem vessels colonized by GFP-expressing hyphae in necrotrophic phase of infection. (Bar = 50 µm). (**s**) Monitoring of disease progression by relative quantification of fungal biomass by q-PCR from DNA extracted from sesame roots infected with *M. phaseolina*. (**t**,**u**) Relative expression of *M. phaseolina* stage-specific marker genes for biotrophic stage (biotrophy associated secreted protein-BAS3) and necrotrophic stage (Necrosis inducing protein-NIP) in infected sesame roots at different time points post inoculation.

The onset of necrotrophic phase was characterized by extensive growth of thin, secondary hyphae which necrosed the host tissues (Fig. 2a,c). The growth of the thin necrotrophic hyphae was intra- and inter-cellular in both varieties (Fig. 2b,d,e,h). The extent of GFP expressing hyphal growth over the root surface was visibly less in Nirmala roots (Fig. 2f) than VRI-1 where the mycelia formed a mantle covering the susceptible root (Fig. 2i). Formation of dark microsclerotia appearing as black spots was visibly less in the resistant variety (Fig. 2g) than the susceptible one (Fig. 2j). This resulted in less external symptoms in Nirmala roots (Fig. 2k) than VRI-1 (Fig. 2m). Cross sections revealed that VRI-1 roots were heavily colonized with fungal hyphae which induced more tissue necrosis (Fig. 2n), compared to Nirmala (Fig. 2l).

After root infection, *M. phaseolina* colonized the lower stem, which externally appeared ashy black (Fig. 2o). Confocal microscopy revealed that the fluorescent hyphae colonized the stem cortical tissue in both varieties (Fig. 2p). Ultimately hyphae clogging the xylem vessels was visible in longitudinal sections (Fig. 2q) and cross sections (Fig. 2r), blocking the water supply, causing plants to collapse in the necrotrophic phase in both varieties.

### Transition from biotrophy to necrotrophy in *M. phaseolina* is confirmed by changes in fungal biomass and expression of fungal biotrophy and necrotrophy marker genes

Our results show that delayed disease progression in Nirmala correlates withless fungal biomass. At 12 hpi, during biotrophic phase in both varieties, no significant differences in the amounts of fungal DNA were detected (Fig. 2s). At later time points, during BNS and necrotrophic phase, fungal proliferation was less in Nirmala compared to VRI-1.

The expression of *M. phaseolina* biotrophy marker gene *BAS3* (Table 1) was consistently higher in Nirmala compared to VRI-1 (Fig. 2t). With the onset of BNS (24 hpi for VRI-1 and 36 hpi for Nirmala), there was a sharp decrease in its expression. The *M. phaseolina* necrotrophy marker gene *NIP* (Table 1) showed gradual increase in expression in VRI-1 during late hours of biotrophy and it was predominantly expressed throughout necrotrophic phase in greater amounts compared to Nirmala (Fig. 2u), correlating with greater necrosis.

**Table 1:** List of primers used in this study for *M. phaseolina* genes.

No.	Gene name	Forward primer (5′-3′)	Reverse primer (5′-3′)	Size of amplicon
**1**.	**BAS3**	GGTGCTTGAAAGACCCGTAA	TACCCATTGGAGCAGAATCC	188
**2**.	**NIP**	AACATCCTTCGTCTCCCAAA	GAACTGCACCTGGTGGTTCT	186
**3**.	**β-Tubulin**	CCTCCATAGGTTCACCTCCA	AGAGATGGTCTGCCTGTGGT	182

### Less ROS accumulation, upregulation of ROS signaling genes and better antioxidant enzyme activities post BNS correlate with resistance

**Figure 3:**
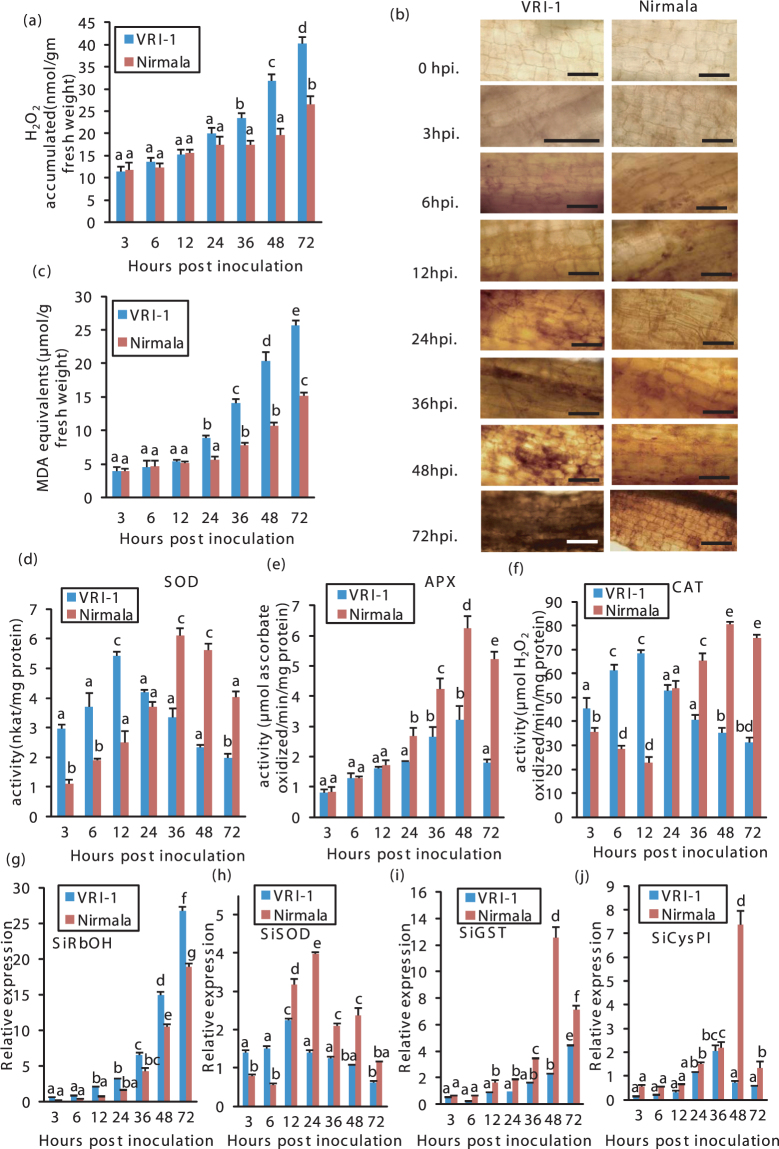
ROS metabolism and anti-oxidant enzyme activity in sesame during different phases of infection in resistant (Nirmala) and susceptible (VRI-1) varieties of sesame. (**a**) Quantitative estimation of H_2_O_2_ accumulation in different hours post inoculation (hpi). (**b**) Micrograph showing H_2_O_2_ accumulation in root cells after infection detected by DAB staining. (**c**) Quantitative estimation of lipid peroxidation (MDA equivalent). Quantitative estimation of enzyme activity of (**d**) superoxide dismutase (SOD) (**e**) ascorbate peroxidase (APX) (**f**) Catalase at different hpi. RT-qPCR analysis of the expression of antioxidant genes (**g**) *SiRbOH* (*Sesamum indicum* respiratory burst oxidase homolog), (**h**) *SiSOD* (*S. indicum* Superoxide dismutase) (**i**) *SiGST* = (*S. indicum* glutathione-s-transferase). (**j**) *SiCysPI* (*S.indicum* Cysteine protease inhibitor). Bars represent standard error (SE) of the mean (n = 3). Different letters indicate significant differences among treatments at *p* < 0.05, according to Duncan’s multiple range test.

Production of ROS is known to be one of the earliest events in response to microbe recognition. At early biotrophic infection phase, no difference was detected in H_2_O_2_ accumulation in the roots of VRI-1 and Nirmala (Fig. 3a). However, with the onset of BNS, after 24 hpi, there was higher accumulation of H_2_O_2_ in VRI-1 roots compared to Nirmala and remained higher until end of experiment (72 hpi) (Fig. 3a). This observation was supported by DAB staining of roots for H_2_O_2_ accumulation (Fig. 3b). During biotrophic phase up to 12 hpi, malonedialdehyde (MDA) content, a measure of lipid peroxidation, was not much different in the two varieties. From 24 hpi onwards which coincided with the BNS phase, there was consistently higher lipid peroxidation in VRI-1 compared to Nirmala (Fig. 3c). In the necrotrophic phase, much less lipid peroxidation was found in Nirmala, owing to successful ROS detoxification, contributing to less necrotrophy-induced tissue damage.

Consistent with the high H_2_O_2_ accumulation, antioxidant activity of SOD was less in Nirmala throughout biotrophic phase compared to VRI-1 (Fig. 3d). An interesting observation was that after 36 hpi i.e. during the necrotrophic phase, antioxidant activity of SOD became higher in Nirmala compared to VRI-1. For APX enzyme, until 12 hpi there was no significant difference in activity in the two varieties after which there was a steady increase in activity in Nirmala which peaked at 48 hpi (Fig. 3e). Catalase showed changes in activity similar to that of SOD during biotrophic to necrotrophic phase shift in the two varieties (Fig. 3f).

**Figure 4:**
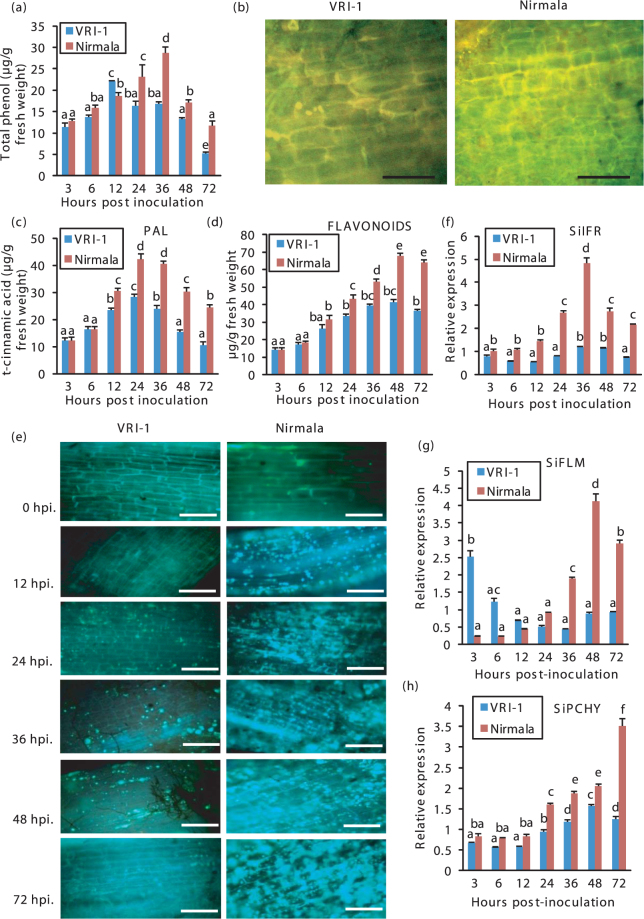
Changes in secondary metabolites of sesame during different phases of infection by *M. phaseolina*. (**a**) Quantitative estimation of total phenol at different hpi. VRI-1 shows peak accumulation of phenolics at 12 hpi where as in Nirmala it the peak was at 36 hpi. (**b**) Micrographs showing less yellow autofluorescence for cell wall bound phenolics in VRI-1 cells than Nirmala at 36 hpi. (**c**) Quantitative estimation of PAL (phenylalanine ammonium lyase) activity at different hpi. (**d**) Quantitative estimation of flavonoids at different hpi. (**e**) Micrograph showing callose deposition in sesame cells at different hpi, with Nirmala showing earlier and more callose deposition than VRI-1. RT-qPCR analysis of the expression of genes for flavonoid biosynthesis: (**f**) *SiIFR* (*S. indicum* Isoflavone reductase) (**g**) *SiFLM* (*S. indicum* Flavonoid 3′-monooxygenase) (**h**) *SiPCHY* (*S. indicum* Paracoumaric-3-hydroxylase). Bars represent standard error (SE) of the mean (n = 3). Different letters indicate significant differences among treatments at *p* < 0.05, according to Duncan’s multiple range test.

Expression analyses of four genes associated with ROS signaling pathway, *SiRbOH*, *SiSOD*, *SiCysPI* and *SiGST* ^19–22^ (Tables 2,3) was done over a time course to get an insight into the regulation of these genes during different phases of infection. *SiRbOH*, a ROS signaling gene, showed steady increase in its expression from late biotrophy to necrotrophic phase, with greater accumulation of transcripts in VRI-1 compared to Nirmala, indicating greater ROS accumulation in susceptible variety (Fig. 3g). In spite of early spiking of ROS scavenging gene *SiSOD* in VRI-1, from 12 hpi onwards Nirmala maintained a constant higher expression, indicating better ROS scavenging (Fig. 3h). Expression of *SiGST*, *SiCysPI* remained similar up to 12 hpi in the two varieties, after which there was gradual increase in the rate of transcript accumulation in Nirmala compared to VRI-1, reaching a peak at 48 hpi (Fig. 4i,j).

**Table 2:** List of genes studied in sesame infected with *M. phaseolina*.

Pathway involved	Acronym of genes used in this study	Function of gene	Genbank accession number
ROS signaling	*SiRbOH*	*Sesamum indicum* Respiratory burst oxidase homolog A	XM_011082597.1
	*SiSOD*	*Sesamum indicum* Superoxide dismutase (Cu-Zn)	XM_011092562.1
	*SiCysPI*	*Sesamum indicum* cysteine proteinase inhibitor A-like (LOC105163741)	XM_011082189.1
	*SiGST*	*Sesamum indicum* glutathione S-transferase	XM_011081394.1
Secondary metabolite	*SiIFR*	*Sesamum indicum Iso*flavone reductase homolog (LOC105172735)	XM_011094294.1
	*SiFLM*	*Sesamum indicum* flavonoid 3’-monooxygenase (LOC105157984)	XM_011074564.1
	*SiPCHY*	*Sesamum indicum* p-coumarate-3-hydroxylase	AY065995.1
Defense-JA-ET signaling	*SiAOS*	*Sesamum indicum* Allene oxide synthase (LOC105178483)	XM_011101964.1
	*SiDef*	*Sesamum indicum* defensin	XM_011085787.1
	*SiSAM*	*Sesamum indicum* S-adenosyl methionine synthase 1 (LOC105168993)	XM_011089234.1
	*SiERF*	*Sesamum indicum* ethylene-responsive transcription factor ERF071 (LOC105163098)	XM_011081322.1
	*SiAP2*	*Sesamum indicum* APETALA 2 (AP2)	KM190074.1
	*SiPDF1.2*	*Sesamum indicum* Plant defensin 1.2	SIN_1021110
	*SiJAZ*	*Sesamum indicum* Jasmonate-ZIM Domain protein	SIN_1026428
SA signaling	*SiEDS1*	*Sesamum indicum* Enhanced disease susceptibility1	SIN_1020515
	*SiNPR1*	*Sesamum indicum* Nonexpressor of PR1	SIN_1007630
	*SiTLP*	*Sesamum indicum* thaumatin like protein	XM_011085956.1
	*SiChi*	*Sesamum indicum* chitinase-like protein 1 (LOC105158761)	XM_011075613.1
Ca^+2^ signaling	*SiCaM*	*Sesamum indicum* Calmodulin (LOC105163726)	XM_011082170.1
	*SiCDPK*	*Sesamum indicum* calcium-dependent protein kinase 32-like (LOC105162537)	XM_011080588.1

**Table 3:** List of primers for sesame genes used in this study.

No.	Gene name	Forward primer (5′-3′)	Reverse primer (5′-3′)	Size of amplicon
**1**.	**SiRbOH**	GAGCTATGGCAGCTGGAAAC	TCCAAGTGAAAAGCCCAATC	229
**2**.	**SiSOD**	TGTACTTTCGGTACCTCAGG	TCTACCTAGTCGGGTGAAAC	172
**3**.	**SiCysPI**	AAACACCATCAGTAGCTGC	CACATACTCCGGTTCCACAC	151
**4**.	**SiGST**	TAGAAGTTTCGGACCCTGGA	ACTACGACAACCAGTCTTCG	183
**5**.	**SiIFR**	CTCGCACGTTGAACAAGACA	CTTCCTCCCCATTTTCCCCT	246
**6**.	**SiFLM**	TTCACAGGGATCGAATGGGT	CGTACTGGAAGTTGTGTCCG	154
**7**.	**SiPCHY**	TACGGCCCCATTTTCTCTGT	AGCCCTTCAAGCCTCTTCAT	245
**8**.	**SiAOS**	CTTCTGGCTCGTCAAATCCG	AGAAGATCAAGAACCCGCCA	170
**9**.	**SiDef**	TCTTTTGTCATCGTTCCGCG	CAACGAAGACGTGGTTTGTG	214
**10**.	**SiSAM**	GTCATCCCAGCCAAGTACCT	TTCTGTCCACCTTAGTCGGG	190
**11**.	**SiERF**	AAACTTCCCAAAGCACTTCC	ACGAAAGTCAACGAGGTTAC	200
**12**.	**SiAP2**	AGGGTTTAGAGAGCACCACC	CTCTGCGCTGCTTCGTTATT	192
**13**.	**SiPDF1.2**	TGCTCTTGGTGTTTGCTTTG	AAACCTTCACAATCGCCATC	151
**14**.	**SiJAZ**	GACTGCTCCCATGACCATTT	CACGACTTTCCTTTCGCTTC	236
**15**.	**SiEDS1**	GATGGATTTGAGGGCAGAAA	AGCCATCTCTGCGTGAATCT	167
**16**.	**SiNPR1**	CTGGGGAATGGTTCTCTTGA	GTGAGATTGCCATTCGGTTT	176
**17**.	**SiTLP**	CGTCATAGACCCCCTCAACT	AATCGCGTAAACGAGGAAAA	202
**18**.	**SiChi**	TTTGGGTACAACTTTAAGCG	GTTGGTCTGCTAAAGCCTAA	187
**19**.	**SiCaM**	CTTGGGGAGAAGCTGACTGA	GTCTGCCCTGACCTCTTTCT	182
**20**.	**SiCDPK**	AAGAGCTGAGGGATGCCTTT	CGATGCTTTTCTCCAGTCCG	150
**21**.	**SiEIF4A**	AGCCCGTCCGCATTCT	AAGCCAGTCAACCTTTCTCC	176

### Greater accumulation of secondary metabolites and upregulation of secondary metabolite-related genes after BNS correlate with resistance

Production of antimicrobial secondary metabolites constituted one of the earliest defense responses in sesame against *M. phaseolina*. Up to 6 hpi, the accumulation of phenolics was similar in the two varieties with in fact more accumulation in VRI-1 at 12 hpi. But at the onset of necrotrophic phase in Nirmala (36 hpi) there was a sudden increase in the rate of accumulation of phenolics (Fig. 4a). This was supported by the detection of stronger autofluorescence of cell wall-bound phenolics in infected Nirmala root cortical cells compared to VRI-1 at 36 hpi (Fig. 4b). Accumulation of phenolics correlated with high PAL activity, the key enzyme in biosynthesis of p-hydroxycinnamic acid and derivative phenolic compounds, in Nirmala throughout biotrophic and necrotrophic phase with a wider gap between Nirmala and VRI-1 after 36 hpi (Fig. 4c). Similar results were obtained for flavonoids (Fig. 4d).

Callose deposition along the cell-wall, which is known to impede hyphal growth, initiated as early as 12 hpi in Nirmala. Time course detection of callose by fluorescent microscopy showed visibly higher amount of callose deposition in Nirmala roots than VRI-1 throughout biotrophic and necrotrophic phase (Fig. 4e). Differential accumulation of secondary metabolites during different phases of infection in the two varieties in mediating better tolerance against *M. phaseolina* in the resistant variety was further confirmed by transcriptional activation of *SiIFR*, *SiFLM* (involved in Isoflavone and Dihydroflavonol biosynthesis respectively) and *SiPCHY* (involved in lignin biosynthesis and structural barrier defense)^23,24^. Expression of *SiIFR* was higher in Nirmala compared to VRI-1throughout biotrophic and necrotrophic phase but a distinct jump in the expression level occurred at BNS in Nirmala (Fig. 4f, Tables 2 and 3). Expression of *SiFLM* was higher in VRI-1 compared to Nirmala during the biotrophic phase but during BNS there was a sudden increase in expression in Nirmala becoming three to four times higher than VRI-1 in the subsequent time points (Fig. 4g). *SiPCHY* transcript accumulation on the other hand was constantly higher in Nirmala throughout the infection phases (Fig. 4h).

### Differential expression of defense signaling genes with respect to changing phases of the pathogen results in coordinated defense response

A total of 13 different genes were selected from four signaling cascades to observe if the host can alter its defense strategy with changing the phases of the pathogen and whether the tolerant host makes more prompt alterations compared to the susceptible host. The acronyms of the genes from different pathways and primers are given in Tables 2, 3.

**Figure 5:**
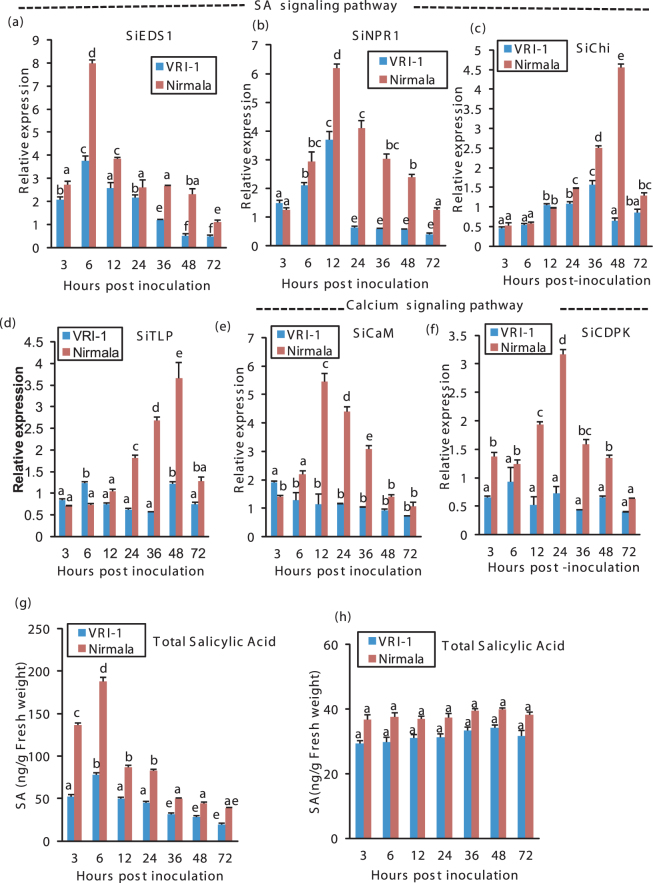
RT-qPCR analysis of the expression of SA, calcium signaling genes and SA estimation in sesame during different phases of infection by *M. phaseolina*. (**a**) *SiEDS1* (*S. indicum* Enhanced disease susceptibility 1) (**b**) *SiNPR1* (*Sesamum indicum* Nonexpressor of PR genes1) (**c**) *SiChi* (*S. indicum* chitinase) (d) *SiTLP* (*S. indicum* Thaumatin like protein) (**e**) *SiCaM* (*S. indicum* calmodulin) (**f**) *SiCDPK* (*Sesamum indicum* calcium dependent protein kinase). Sesame *eIF4A* was used as an internal control. (**g**) GC-MS quantification of SA in the two varieties post inoculation with *M.phaseolina*. (**h**) GC-MS quantification of SA in the two varieties under mock inoculation. Bars represent standard error (SE) of the mean (n = 3). Different letters indicate significant differences among treatments at *p* < 0.05, according to Duncan’s multiple range test.

Upregulation of SA-inducible genes, as consequence of SA buildup contributes to early defense response after pathogen recognition during biotrophic phase. Early peaking and consistent greater transcript accumulation of SA regulated genes (*SiEDS1, SiNPR1*) in Nirmala compared to VRI-1 contributes to early immune response (Fig. 5a,b). Again, SA-regulated PR genes *SiTLP* (PR-5), *SiChi* (PR-3) showed higher expression in the late biotrophic phase and BNS for defense against biotrophic phase and then extending into the necrotrophic phase likely for inducing SAR (Fig. 5c,d).

SA levels were directly quantified to see if SA levels increased with activation of SA-regulated genes. SA levels peaked at 6 hpi and showed greater accumulation in the biotrophic phase and thereafter decreased in the necrotrophic phase (Fig. 5g). SA accumulation was overall greater in Nirmala. Under mock inoculation there was no significant difference in SA level between two varieties (Fig. 5h).

Sesame Calmodulin homolog, *SiCaM* (regulator of Ca^+2^ signal transduction) expression was higher during the BNS phase in Nirmala. In VRI1 *SiCaM* expression remained at constant low level compared to Nirmala (Fig. 5e). *SiCDPK* (which sense intracellular Ca^+2^ levels and translates signal to phosphorylation events) also showed a peak just prior to BNS in Nirmala but in VRI-1 the level was constant (Fig. 5f). Participation of *SiCaM* and *SiCDPK* in early response to pathogen infection indicates role of intracellular Ca^+2^ signaling in activation of defense in sesame just prior to BNS of pathogen.

**Figure 6:**
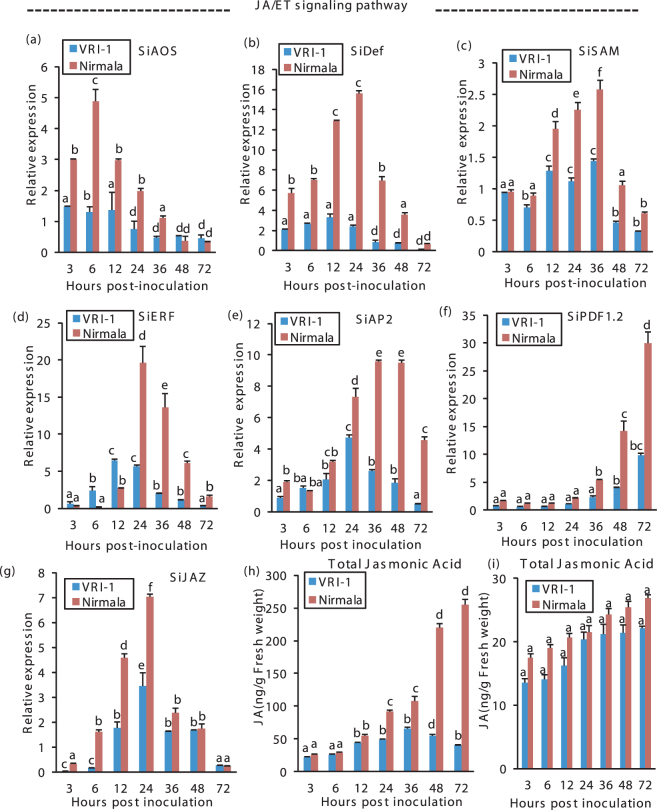
RT-qPCR analysis of the expression of JA/ET signaling genes and JA estimation in sesame during different phases of infection by *M. phaseolina*. (**a**) *SiAOS* (*S. indicum* Allene oxide synthase) (**b**) *SiDef* (*Sesamum indicum* Plant defensin) (**c**) *SiSAM* (*S. indicum* S-Adenosyl methionine synthetase) (**d**) *SiERF* (*S. indicum* Ethylene response factor) (**e**) *SiAP2* (*S. indicum* EREBP/Apetalla2) (**f**) *SiPDF1.2* (*Sesamum indicum plant defensin 1.2*) (**g**) *SiJAZ* (*S. indicum Jasmonate ZIM-domain* protein). Sesame *eIF4A* was used as an internal control. (**h**) GC-MS quantification of JA in the two varieties post inoculation with *M*. *phaseolina*. (**i**) GC-MS quantification of JA in the two varieties under mock inoculation. Bars represent standard error (SE) of the mean (n = 3). Different letters indicate significant differences among treatments at *p* < 0.05, according to Duncan’s multiple range test.

With approach of the necrotrophic phase the activation of JA-ET signaling was evident (Fig. 6a–h, Tables 2 and 3). Early peaking of *SiAOS* (JA biosynthesis gene) at 6 hpi enabled the plant to promptly upregulate the downstream genes in the JA pathway. Upregulation of *SiSAM* (Ethylene biosynthesis gene) during BNS (36 hpi) in Nirmala indicated activation of JA-ET biosynthesis pathway as consequence of BNS in pathogen. Higher accumulation of marker genes for JA signaling (*SiPDF1.2*, *SiAP2*) and ET signaling (*SiERF*) around BNS and necrotrophic phase distinctly indicates change in defense strategy with respect to changing phases of the pathogen. *SiDef*, another JA regulated gene, peaked at 24 hpi in Nirmala which has a late BNS but at 12 hpi in VRI-1 correlating with earlier BNS in VRI1. *SiJAZ* gene, negative regulator of JA signaling, behaved in opposite way, i.e. showing higher expression in biotrophic phase, with sharp decrease during necrotrophy. JAZ expression trend was similar in Nirmala and VRI-1 but the level was much less in VRI1 (Fig. 6g).

JA buildup was confirmed by quantitative estimation (Fig. 6h). Higher accumulation of endogenous JA in Nirmala around the time of BNS and during necrotrophic correlate with expression pattern of JA-ET regulated genes with respect to the infection stages of the pathogen. Under mock inoculation there was no significant difference in JA content between the two varieties (Fig. 6i).

### Tolerance of sesame against charcoal rot results from activation of JA-ET signaling studied through priming with phytohormones and their inhibitors

**Figure 7:**
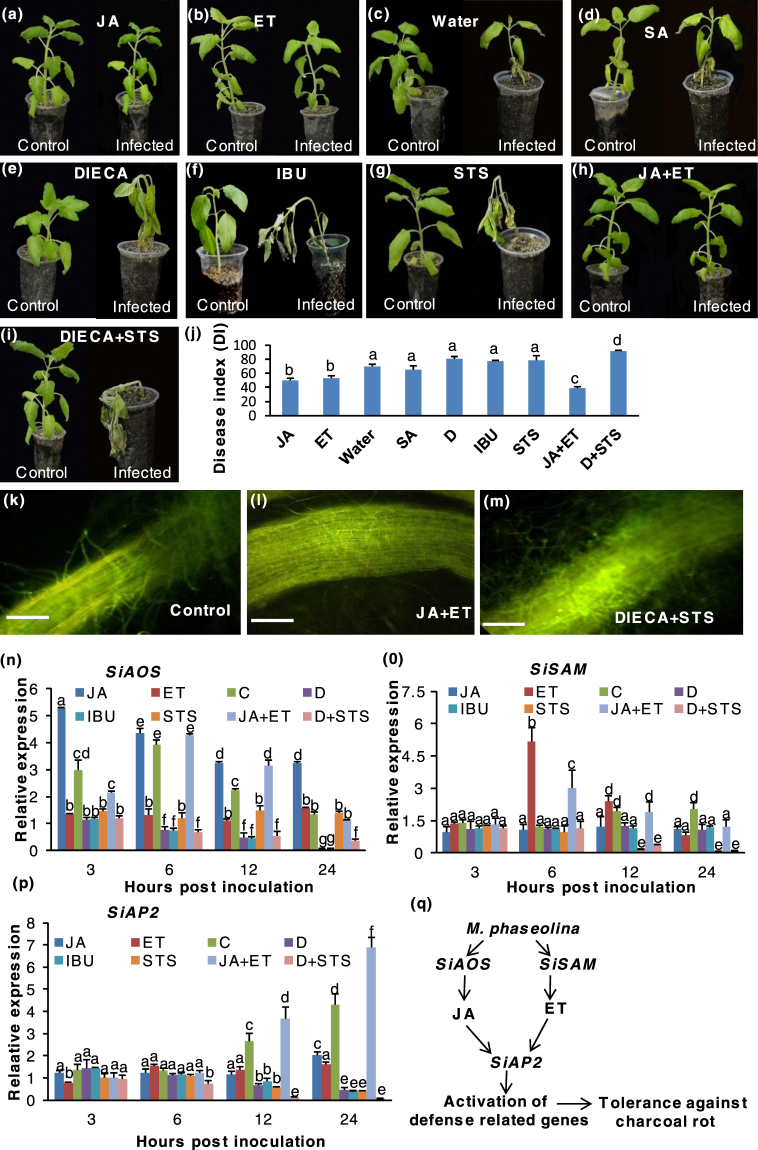
Phytohormone priming experiments by treatment of sesame (resistant variety Nirmala) with different phytohormones and their inhibitors to assay effect on tolerance against *Macrophomina phaseolina*. Plants treated with different chemicals 24 hrs prior to infection with *M. phaseolina* and pictures were taken 14 days post inoculation. (**a**) 100 µM Methyl jasmonate (**b**) 500 µM Ethepon (**c**) water (**d**) 200 µM salicylic acid (**e**) 100 µM DIECA (**f**) 20 µM Ibuprofen (IBU) (**g**) 20 mM STS (**h**) 100 µM Methyl jasmonate + 500 µM Ethepon (**i**) 20 mM STS + 100 µM DIECA (**j**) Disease index for inoculated sesame under different chemical treatment. Bars represent standard error (SE) of the mean (n = 3). Different letters indicate significant differences among treatments at *p* < 0.05, according to Duncan’s multiple range test. Confocal micrograph showing sesame roots infected with GFP-expressing *M. phaseolina* in (**k**) control (**l**) pre-treated with100 µM Methyl jasmonate + 500 µM Ethepon. (**m**) Pre-treated with 20 mM STS + 100 µM DIECA. RT-qPCR analysis of the expression of (**n**) *SiAOS* (**o**) *SiSAM* (**p**) *SiAP2* under different treatments. (**q**) Schematic representation of proposed role of the sesame JA/ET genes in defense activation during *M. phaseolina* infection.

Given the observed activation of phytohormone regulated defense genes upon fungal infection, we investigated the effect of priming with JA/ET/SA and their inhibitors on resistance of sesame (using resistant variety Nirmala) against *M. phaseolina* (Fig. 7a–i). Priming studies showed that in comparison to control (69.93%), no significant difference in Disease index (DI) was observed for SA treated seedlings (65.53%) (Fig. 7j). But for either JA or ET treated seedlings, DI was significantly reduced to 50 and 53.3% respectively (Fig. 7j). When JA and ET were applied together, DI reduced to 39% indicating strong induction of basal defense against *M. phaseolina*. Application of Ibuprofen (JA biosynthesis inhibitor), DIECA (JA pathway inhibitor) or STS (ET pathway inhibitor) alone increases DI to 79.23%, 80% and 77.8% respectively. Combined application of DIECA and STS resulted in high DI (91.11%) indicating intact JA/ET signalling pathway is required for tolerance against charcoal rot (Fig. 7j). This hypothesis has been further confirmed by microscopic study of infected roots of treated plants and control. In plants pre-treated with JA + ET, less hyphal growth was seen on roots compared to control (Fig. 7k,l). In DIECA + STS treated plants, compromised plant defense was correlated with overgrowth of *M. phaseolina* hyphae in roots, resulting in necrosis (Fig. 7m).

Functional activation of JA-ET signaling in imparting charcoal rot tolerance was confirmed by transcriptional analysis of genes involved in the pathway. Activation of JA and ET biosynthetic pathway genes (*SiAOS* and *SiSAM* respectively) under phytohormone treatment at early biotrophic phase is congruent with that in the control set (plants infected with *M. phaseolina* only) thereby indicating involvement of JA-ET signaling pathway to boost early defense responses (Fig. 7n,o). Positive regulation of *SiAOS* and *SiSAM* by JA + ET and suppression of their responses via potent inhibitor of the pathways (DIECA, IBU for JA, STS for ET, DIECA + STS for JA + ET signaling) further supports this notion. Gradual upregulation of *SiAP2* by *M. phaseolina* in control set followed similar expression profile as that observed after application of JA + ET. Either JA or *ET al*.one only slightly increased *SiAP2*, but in much less amount compared control. Application of STS + DIECA (inhibitor of JA, ET pathway) progressively downregulated *SiAP2* expression, thereby further indicating possible regulation of the gene by combined JA-ET signaling (Fig. 7p). Thus it can be inferred that *SiAP2* likely acts downstream of *SiAOS* and *SiSAM* by integrating signals from both pathways (Fig. 7q).

Similar observations were made with priming experiments using VRI1 but as expected the DI was higher (Fig S1 a–j). The expression of *SiAOS*, *SiSAM* and *SiAP2* remained constant up to 24 hrs after mock inoculation (Fig. S1 k,l,m).

## Discussion

The general perception is that the successful establishment of all necrotrophic fungi in a host entails rapid pathogen-mediated host-cell death as the primary step. Recent study with archetypal necrotrophic fungus *Botrytis* has revealed its versatility to switch from symptomless, biotrophic lifestyle to obligate necrotrophy under appropriate conditions^25^. Other studies on previously designated necrotroph such as *Sclerotinia sclerotiorum*, show that the fungus has a brief 24 hrs. biotrophic phase prior to the necrotrophic phase entailing the fungus to be repositioned as a hemibiotroph^25,26^. Therefore, it is possible some of the necrotrophs may have a short biotrophic phase. Thus far *M. phaseolina* has been considered as a broad host-range necrotroph infecting most species of dicot plants^15^. In the present study, a previously undocumented biotrophic phase lasting for a significant period of up to 36 hours in the resistant variety and the transitional phase to necrotrophy has been confirmed in *M. phaseolina*. Along with microscopic observation, time wise transcriptional activation of BAS3 and NIP (stage specific proteins of *M. phaseolina*) distinctly marks the onset of biotrophic and necrotrophic infection phase, similar to other hemibiotroph like *Phytopthora* ^4^. In hemibiotroph *Septoria tritici*, phase transition from symptomless biotrophy to destructive necrotrophy has been directly correlated with increase in fungal biomass^10^ which is in congruence with transcriptional data from current study, indicating the hemibiotrophic nature of *M. phaseolina*. The symptomless biotrophic phase of the charcoal rot fungus is a cause for concern under field conditions as this could make the disease virtually undetectable during early stages of infection. Since physiological switching in lifestyle of a pathogen entails an ecologically flexible outcome in terms of disease progression^27^, it is important that based on our findings *M. phaseolina* be repositioned among hemibiotrophs rather than necrotrophs.

Accumulation of ROS, such as H_2_O_2_, may also have contrasting defense functions depending on a pathogen’s lifestyle. While the level of ROS accumulation acts as virulence factor for disease development in necrotrophs like *B. cinerea*, *Alternaria solani* ^28^ on other hand the production of ROS, at the penetration site is one of the earliest defense reactions to resist fungal growth and is particularly useful against biotroph^9^. For hemibiotroph *S. sclerotiorum*, in the early stage, pathogen creates a reducing environment that suppresses the oxidative burst, but later it utilizes ROS induced cell death for successful infection^29^. In hemibiotroph *Septoria tritici*, *Verticillium dahliae*, infection is characterized by massive H_2_O_2_ accumulation during necrotrophy. RbOH (Respiratory burst oxidase homologs) in plants are directly involved in ROS accumulation and plant susceptibility against necrotrophic fungi^30^. Disease tolerance against hemibiotrophic *F. oxysporum* has been co-related in resistant banana with reduced expression of an RbOH homolog compared to susceptible one^31^. Earlier reports show that higher activities of ROS scavenging enzymes from 24 hpi onwards in the necrotrophic phases confer better ROS homeostasis in resistant varieties^32,33^. In present study, less H_2_O_2_ accumulation in Nirmala through reduced expression of RbOH and higher activity of ROS scavenging enzymes corresponds to delayed disease progression and tolerance, which is in line to a recent study in *Medicago truncatula*, where reduced ROS accumulation in roots show enhanced resistance to root-rot pathogen *Aphanomyces euteiches* ^28^.

Isoflavonoids, flavonoids or phenylpropanoid derivatives are some of the widely used secondary metabolites employed in plant immunity^24,34^. Activation of phenylpropanoid pathway as part of defense response in sesame against *M. phaseolina* was evidenced earlier by polyphenol accumulation and increased PAL activity^35^. The significantly higher PAL activity in resistant Nirmala from 12 hpi onwards as basal immune response, led to the accumulation of both free and cell wall-bound phenolics similar to that reported during the hemibiotroph *Colletotrichum* infection in maize^9^. Activation of phenylpropanoid pathway led to flavonoid biosynthesis, as estimated by higher *SiIFR* and *SiFLM* expression, similar to defense response in wheat against hemibiotroph *Fusarium graminearum* ^12^. Defense response also included lignin biosynthesis, as indicated by *SiPCHY* peaking at necrotrophic phase in both varieties of sesame. Consistent higher callose deposition in Nirmala led to less necrosis-induced tissue damage compared to VRI-1 by increased cell wall reinforcement, consistent with earlier report on wheat^36^ Overall the higher activity of secondary metabolite pathway in resistant variety is one of the key factors behind its tolerance against *M. phaseolina*.

For hemibiotrophic pathogen, plant defense is dependent on coordinated and orderly expression of SA, JA signaling^37,38^ although response is differential between susceptible and resistant genotype^39^. RNA seq analysis in *Brassica* showed activation of SA signaling during biotrophic infection of *Leptosphaeria*, which shifts to JA signaling during necrotrophy^40^. In this study we found we found significant increase in SA level during biotrophy and JA during necrotrophy which correlates with transcript accumulation. Such results are in accordance to recent study in tomato infected with hemibiotroph *Phytopthora* ^41^, thereby directly indicating involvement of both phytohormones during different infection stage. Similarly, Ca^2+^ signaling is critical for transcriptional reprogramming in plant’s innate immunity^42^. In response to *M. phaseolina* infection, *SiCaM* and *SiCDPK* were rapidly but coordinately induced in Nirmala during early hours of biotrophic infection phase with gradual reduction after 24 hpi. This is consistent with that observed in other plants that there is transient Ca^2+^ accumulation in the cytoplasm during the initial plant-pathogen interaction^43^.

For long lasting activation of induced resistance in plants, modulation of immunogenic memory with a low, non-effective concentration of a phytohormone gives better, faster and stronger immune response than that in non-primed plants^44^. Upon priming plants are capable of enhancing their basal resistance against future pathogenic attack by either improving perception of pathogen signals or by systemic activation of defense related pathways^45^. Following microbe perception, plants produce a complex blend of JA, ET and SA with specific combination seemingly depending on the infection strategy and lifestyle of the invading pathogen. Our study showed priming using combined JA and ET resulted in enhanced tolerance in sesame against *M. phaseolina* which is similar to an earlier study in *Medicago* where JA + ET application induced partial tolerance against *M. phaseolina* ^46^.As exogenous MeJA application in wheat provides resistance against hemibiotroph *Fusarium* by activation of JA signaling^47^, our results suggests combined JA-ET application prior inoculation is likely contributing to plant immunity by synergistic activation of JA-ET signaling in advance to fungal infection^48,49^.

Synergism between the defense activation pathways controlled by JA and ET is mainly characterized by the induction of *PDF1.2* via *ERF1* and *ORA59*, which acts as integrator of two signaling pathway^49,50^. Pharmacological inhibitor and transcriptional study showed *SiAP2* (member of ERF family), downstream of both *SiAOS* and *SiSAM*, showed highest expression under combined JA/ET treatment followed similar pattern as induced by *M. phaseolina*. Our results suggest probable involvement of *SiAP2* as integrator of both JA, ET signaling which supposedly activates upon *M. phaseolina* infection as evident by transcriptional upregulation of JA, ET biosynthesis genes. JA and ET independently activate not just separate sets of genes, they also synergistically activate defensins like *PDF1.2* and PR genes^49^. Activation of *SiPDF1.2*, *SiTLP* and *SiChi* during necrotrophic indicates *SiAP2* effectively upregulate downstream defense signaling genes, thereby leading to tolerance in sesame against charcoal rot.

In this article we present a comprehensive study of the host pathogen interaction between the hemibiotrophic pathogen *M. phaseolina* with a resistant and a susceptible variety of sesame. For successful infection, the hemibiotrophic fungi utilize biotrophy to necrotrophy switch so as to trick the host into applying PCD during the initial biotrophic phase. This adaptation enables the fungus to take full advantage of defense-related cell death during the subsequent necrotrophic stage, as seen in some recent studies^11^. Following pathogen perception, plants are known to produce complex and layered defense response seemingly depending on the infection strategy and lifestyle of the invading pathogen^37^. Here, just as the pathogen changed its strategy of infection, switching from biotrophic to necrotrophic lifestyle, the host could tailor its defense strategy to meet the changing situation. Most importantly this defense response was more prompt in the resistant host compared to the susceptible host, signifying that a resistant variety makes different choices from a susceptible host during the changing infection stages which influences the outcome in terms of disease progression. Our findings uncover new areas of research that will allow a better understanding of hemibiotrophic interactions and hosts responses.

## Materials and Methods

### Plant maintenance, fungus inoculation and molecular characterization of disease development

Susceptible (VRI-1) and resistant (Nirmala) varieties of sesame^15^ were grown aseptically in Hoagland’s agar medium. Pure culture of *M. phaseolina* was maintained according to our publication^15^. For fluorescence microscopy, *M. phaseolina* was transformed with GFP constructs via *Agrobacterium*-mediated method according to our protocol^51^. For infection studies, PDA discs of the fungalculture were placed in Hoagland’s agar medium and allowed to grow. At the edge of actively growing hyphae, roots of six week old sesame seedlings were placed, allowing fungus to infect. Root infection was periodically monitored at different time points (hours post inoculations, hpi) by bright field microscopy, epifluorescence microscopy (Leica *DMLS*, Germany) and confocal microscopy (Olympus, Singapore). To define the infection phases of *M. phaseolina* within host, expression analysis of marker genes for biotrophy (**BAS3**, Biotrophy associated secreted protein-EKG18082.1) and necrotrophy (**NIP**, Necrosis inducing protein-EKG16185.1) were analyzed by q-RT-PCR. *M.phaseolina* β-tubulin gene (KF952242.1) was used as an internal control for normalization of expression. List of primers for these genes are in Table 2. Fungal biomass was quantified by determination of fungal DNA in plant extract by q-PCR using primers *mp*(f) 5′-CCGAAGAATATCCACCTCCA-3′ and *mp*(r) 5′-GCAAAGGGTGGAAAGGAAGT-3′ spanning internal transcribed sequences of rRNA gene (JN241996.1)^52^. For normalization, the sesame *eIF4A* was amplified with primers *eIF4A*(f) 5′-AGCCCGTCCGCATTCT-3′ and *eIF4A(r)* 5′*-*AAGCCAGTCAACCTTTCTCC-3′

### Calculation of Disease index

Disease index was calculated according to our earlier publication^15^, Chowdhury *et al*., 2014.

### Analysis of gene transcript level by reverse transcription-quantitative real time PCR (RT-qPCR)

Isolation of RNA and cDNA synthesis was performed according to our published protocol^53^. RT-qPCR reactions were carried out in 20 µl volume, 96-well blocks, (Applied Biosystems 7500 Real-Time PCR System, USA) and the Power SYBR Green mastermix (Applied Biosystems). The sesame genome sequence^17^ was used to identify candidate genes, known to act in different signaling pathway in other plants and were ascribed with acronyms which were maintained throughout the study (Table 1). Primers for the selected genes were designed using Primer-3plus-software (http://www.bioinformatics.nl/cgi-bin/primer3plus/primer3plus.cgi) and IDT-oligo-analyzer 471 (http://www.idtdna.com/analyzer/Applications/OligoAnalyzer/). Amplified genes were sequenced to confirm their identity (Table 2). According to our publication^54^ Sesame *eIF4A* gene was used as an internal control to normalize the variations in the cDNA amounts used for RT-qPCR. All RT-qPCR reactions were performed in biological triplicates using RNA samples extracted from three independent plants grown under identical conditions. The comparative method (ΔΔCt) was employed to evaluate the relative quantity of each amplified product in the samples. The specificity of the PCR reactions was determined based on a melting curve analysis of the amplified products using the standard method installed with the system.

### Analysis of ROS, lipid peroxidation and antioxidative enzyme activities

Hydrogen peroxide (H_2_O_2_) was quantified using Amplex red according to Chakraborty^55^ and histochemical detection in roots was visualized by DAB staining. Lipid peroxidation was estimated according to Chowdhury^56^. Antioxidant enzyme assays were done accordingly.

#### SOD

Enzyme extract was prepared by homogenizing tissue in 0.1 M Sodium Phosphate buffer (pH = 7). After centrifugation, supernatant was used for enzyme assay. The reaction mixture was prepared by mixing 1.110 ml of 50 mM phosphate buffer (pH 7.4), 0.075 ml of 20 mM L-methionine, 0.040 ml of 1% (v/v) Triton X-100, 0.075 ml of 10 mM hydroxylamine hydrochloride and 0.1 ml of 50 µM EDTA. To this mixture 100 µl of enzyme extract and 80 µl of riboflavin (50 µM) were added. The reaction mixture was mixed and then illuminated for 10 min under two 20 W-Philips fluorescent lamps (60 µmol m^−2^ s^−1^). After 10 min exposure, 1 ml of Greiss reagent (prepared freshly by mixing equal volume of 1% sulphanilamide in 5% phosphoric acid and 0.1% N-1-napthyl ethylene diamine) was added and the absorbance was measured at 543 nm. The activity was calculated as nkat/mg protein

#### APX

Enzyme extract was prepared by homogenizing tissues in 50 mM potassium phosphate buffer (pH 7.8) containing 1.0 mM EDTA, 1% PVP, 1 mM ascorbic acid, and 1 mM phenylmethylsulfonyl fluoride at 4 °C. Reaction mixture in a final volume of 3 ml contained 50 mM potassium phosphate buffer (pH 7.0), 0.2 mM EDTA, 0.5 mM AsA, 0.2 mM H_2_O_2_, and the enzyme at 25 °C. H_2_O_2_ was the last component to be added and the rate of ascorbate oxidation was monitored by the decrease in absorbance at 290 nm (extinction coefficient of 2.8 mM^−1^ cm^−1^) up to 5 min. Specific activity of enzyme was expressed as μmol ascorbate oxidized min^−1^ mg^−1^ protein.

#### Catalase

Enzyme extract was prepared by crushing infected roots in 50 mM Tris–NaOH buffer (pH 8.0) containing 0.5 mM EDTA, 2% (w/v) polyvinyl pyrrolidone (PVP), and 0.5% (v/v) Triton X-100 and after centrifugation supernatant was used for enzyme assay. The assay mixture contained 100 mM potassium phosphate buffer (pH 7.0), 50 mM H_2_O_2_, and 200 μl enzyme extract in a final volume of 3 ml. The decomposition of H_2_O_2_ was followed at 240 nm (extinction coefficient of 0.036 mM^−1^ cm^−1^) by decrease in absorbance for 5 min at 25 °C. Catalase activity was expressed as μmol of H_2_O_2_ oxidized min^−1^ mg^−1^ protein.

### Determination of phenolics, flavonoids, Phenylalanine ammonium lyase (PAL) activity and callose deposition

Phenolics and flavonoids from infected root tissue were estimated according to our published paper^33^. Cell wall bound phenolics were detected as bright autofluorescence under epifluorescence microscope (excitation at 436 nm, emission 470 nm)^9^. PAL activity was estimated according to Jogaiah^57^ using trans-cinnamic acid as a standard. The enzyme activity was expressed as µg trans-cinnamic acid/g FW. Fungus-induced callose deposition in infected root tissues were visualized as bluish-green fluorescence by aniline blue staining under epifluorescence microscope (Leica DMLS, excitation maximum 330–385 nm, dichroic mirror DM 400, barrier filter >420 nm). For each time point four roots were examined and in each root, 10 microscopic fields were studied for callose deposition.

### Quantification of phytohormones SA and JA

Quantification of SA, JA from infected root tissues was done by gas chromatography following modified vapor phase extraction method according to Mishina and Zeier^58^. 200 mg of infected root tissue were homogenized with 600 mL of extraction buffer (water:1-propanole:HCl 51:2:0.005), followed by addition of internal standards (D4-SA, dihydrojasmonic acid- 100 ng each) and 1 ml of methylene chloride. The phase separation was done by centrifugation and lower organic phase was dried over Na_2_SO_4_, treated with 2 ml of 2 M trimethyl silyldiazomethane in hexane (Sigma-Aldrich) for 5 min at room temperature to convert carboxylic acids into their corresponding methyl esters. Methylation was terminated with 2 M acetic acid in hexane and the sample was subjected to a vapor-phase extraction using a volatile collector trap packed with Super-Q absorbent (VCT-1/4 × 3-SPQ; Analytical Research Systems). The final evaporation temperature was set to 200 °C, and samples were eluted from the collector trap with 1 ml methylene chloride. Finally, the sample mixture was separated on a gas chromatograph (GC 6890N; Agilent Technologies) equipped with a fused silica capillary column combined with a 5975 mass spectrometric detector (Agilent Technologies). For quantitative determination of metabolites, peaks originating from selected ion chromatograms were integrated. The area of a substance peak was related to the peak area of the corresponding internal standard (SA/D4-SA; JA/dihydro jasmonic acid).

### Priming sesame plants with exogenous phytohormones and chemicals

Priming experiments were done using both susceptible (VRI-1) and resistant (Nirmala) plants. For tolerance assays, 6 week old seedlings were pre-treated with chemicals and at 1 day after treatment were inoculated with *M. phaseolina* by infected soil method according to Kamalkannan^59^. For priming experiments following chemicals were used: MeJA (Sigma-Aldrich), Ethepon (Sigma-Aldrich), salicylic acid (Himedia, India) Silver thiosulphate (STS, inhibitor of ET signaling), Ibuprofen (IBU, Sigma- inhibitor of JA signaling), diethyldithiocarbamic acid (DIECA, SRL-inhibitor of JA signaling). STS was prepared fresh by mixing 100 mM AgNO_3_ and Na_2_S_2_O_3_. Different chemicals were used at following concentrations: MeJA (100 µM), Ethepon (500 µM), Salicylic acid (200 µM), STS (20 mM), DIECA (100 µM), Ibuprofen (20 µM). 15 ml of chemicals (single/combination) were added to plant roots in soilrite (mixture of horticulture-grade expanded perlite, Irish Moss Peat, and exfoliated vermiculite in the ratio 1:1:1) and pots were kept in convirons (28 ± 1 °C, 16/8 hrs photoperiod, light intensity 50 μmol m^−2^ s^−1^ using cool-white fluorescent tubes) for 24 hrs prior to infection. The experiment was done thrice with identical results, using 6 plants of same age for each chemical treatment. Disease development assessment and disease index was calculated according to our earlier published work^15^ after two weeks of inoculation. For transcriptional studies, roots of six week old seedlings were treated with chemicals and after specific time point, tissue was collected for RNA analysis. Roots infected with *M. phaseolina* served as control.

### Statistical analysis

All data are mean of three independent experiments ± S.E.M. Each experiment was done in a completely randomized design (CRD) with three replicates^60^. The data were subjected to one-way analysis of variance (ANOVA) and different letters indicate significant differences between treatments at *p* < 0.05, according to Duncan’s multiple range test (DMRT) using software package for statistical analysis (SPSS version 16, 2007). Data represented are means ± standard error of mean (S.E.M) of three independent experiments with three replicates.

## Electronic supplementary material

Supplemental Figure S1
