# Supplementary material for: Biotrophy-necrotrophy switch in pathogen evoke differential response in resistant and susceptible sesame involving multiple signaling pathways at different phases
Source: Sci Rep. 2017 Dec 8;7:17251. doi: 10.1038/s41598-017-17248-7 (PMC5722813; doi:10.1038/s41598-017-17248-7)
Supplement: Supplementary file 1 — Supplemental Figure S1 [file 41598_2017_17248_MOESM1_ESM.pdf]

Biotrophy-necrotrophy switch in pathogen evoke differential response in resistant and susceptible sesame involving multiple signaling pathways at different phases

Supriyo chowdhury, Arpita Basu, Surekha Kundu

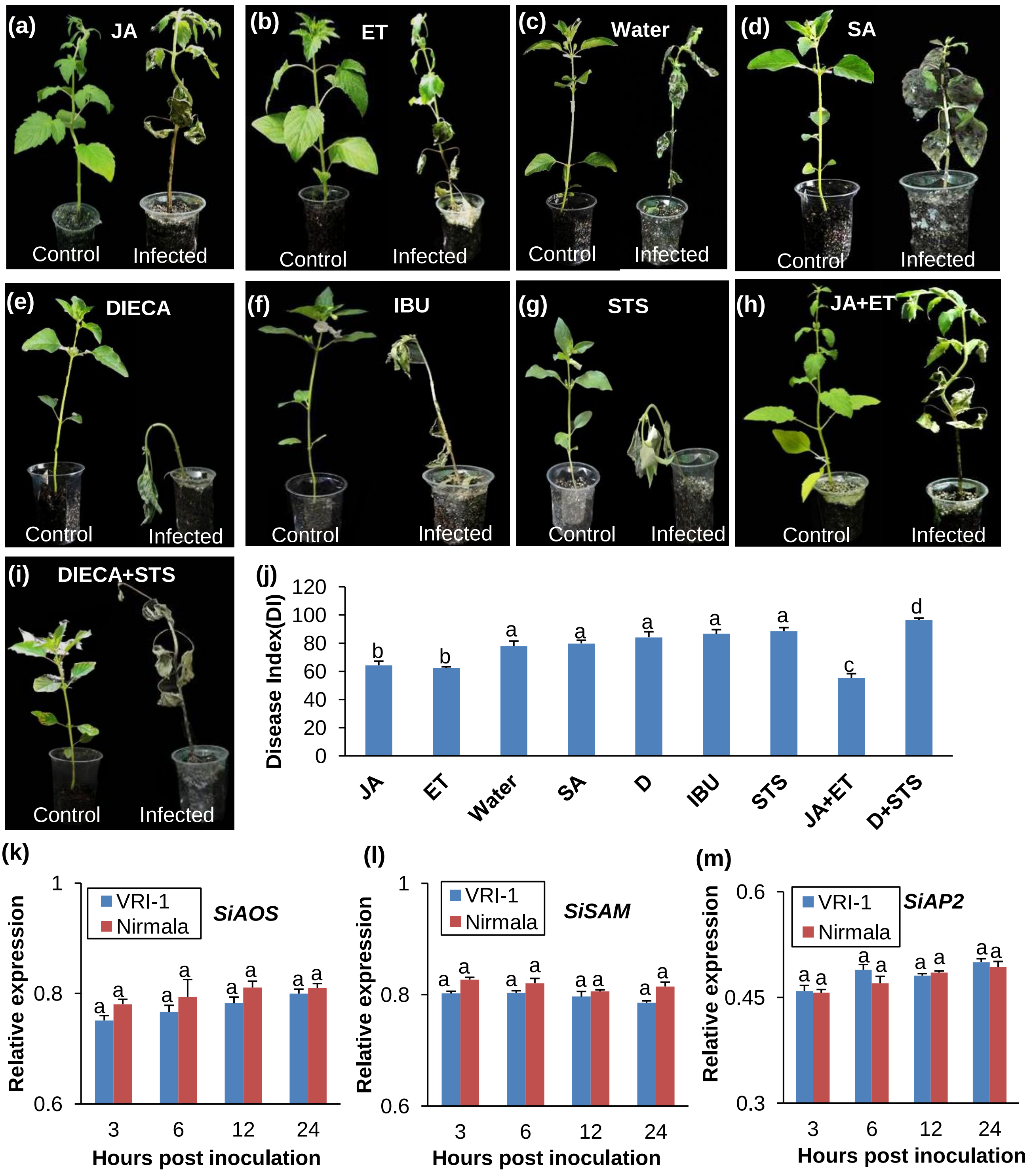

Supplemental Figure S1

Supplemental figure S1: Phytohormone priming experiments by treatment of s esame (susceptible variety VRI-1) with different phytohormones and their inhibitors to assay effect on tolerance against *Macrophomina phaseolina*. Plants treated with different chemicals 24hrs prior to infection with *M. phaseolina* and pictures were taken 14 days post inoculation. (a) 100µM Methyl jasmonate (b) 500µM Ethepon (c) water (d) 200µM salicylic acid (e) 100µM DIECA (f) 20µM Ibuprofen (IBU) (g) 20mM STS (h) 100µM Methyl jasmonate +500µM Ethepon (i) 20mM STS+100µM DIECA (j) Disease index for inoculated sesame under different chemical treatment. RT-qPCR analysis of defense genes of sesame (k) *SiAOS* (l) *SiSAM* (m) *SiAP2* under mock inoculation in susceptible and resistant varieties. Bars represent standard error (SE) of the mean (n = 3). Different letters indicate significant differences among treatments at $p < 0.05$, according to Duncan's multiple range test.
